# Ethnomedicinal Survey of Plants Used for Treatment of Mild COVID-19-Related Symptoms in Gorontalo Province, Indonesia

**DOI:** 10.1155/sci5/5849854

**Published:** 2025-04-21

**Authors:** Wiwied Ekasari, Retno Widyowati, Neny Purwitasari, A. Mu'thi A. Suryadi, Ram K. Sahu

**Affiliations:** ^1^Department of Pharmaceutical Sciences, Faculty of Pharmacy, Universitas Airlangga, Surabaya, East Java, Indonesia; ^2^Institute of Tropical Disease, Universitas Airlangga, Surabaya, East Java, Indonesia; ^3^Department of Pharmacy, Faculty of Sports and Health, Universitas Negeri Gorontalo, Gorontalo, Indonesia; ^4^Department of Pharmaceutical Sciences, Hemvati Nandan Bahuguna Garhwal University (A Central University), Chauras Campus, Tehri Garhwal 249161, Uttarakhand, India

**Keywords:** COVID-19, ethnomedicinal survey, medicinal plant

## Abstract

This study aims to document plant species used to fight several diseases related to mild COVID-19 symptoms such as cough, colds, fever, dizziness and diarrhoea in Gorontalo Province, eastern Indonesia. Data were collected from 105 local residents using semistructured questionnaires, open interviews and field surveys. Ethnobotanical indices including use reports (UR), relative frequency of citation (RFC) and fidelity level (FL) were used to analyse the data. A total of 82 plant species from 40 families were recorded. *Plectranthus scutellarioides* (L.) R.Br. is predominantly used to treat cough, *Zingiber officinale* Roscoe to treat colds, *Bryophyllum pinnatum* (Lam.) Oken to treat fever, *Allium sativum* L. to treat dizziness and *Psidium guajava* L. to treat diarrhoea. The current research reveals that *P. guajava* L. is the most popular plant species with a high index value (RFC 0.62, FL 62%). Leaves (46.46%) are the most widely used part of the plant for some of these diseases. Boiling (31.70%) is the main method in preparing medicinal plants, most of which are given orally (77.73%). In addition, no serious side effects caused by the consumption of these medicinal plants in the treatment of several diseases related to mild COVID-19 symptoms have been reported. Current studies reveal the wealth and wide use of plant species to manage cough, colds, fever, dizziness and diarrhoea in the study area. Traditional knowledge of medicinal plants used by local residents of Gorontalo Province may help in the treatment of several diseases related to mild COVID-19 symptoms. Further research on preclinical studies needs to be conducted to provide scientific evidence of this result.

## 1. Introduction

The COVID-19 pandemic that has occurred to this day has caused global socioeconomic disruption with alarming numbers and health problems [1]. There is no absolute therapy or specific treatment for COVID-19 yet, but infection prevention and control measures have been used to control further complications [2]. Many research trials have been conducted, both single and combined drug use, but have not yielded satisfactory results.

Some natural ingredients can be considered a safe, secure and reliable source for finding medicines that can control the current pandemic. The use of traditional medicines from natural ingredients is one of the potential options to prevent COVID-19 infection [3]. The patient's immune status plays an important role in COVID-19 infection, and traditional drugs that have an immunomodulatory effect can potentially be used as a preventive and even a therapeutic agent for patients with COVID-19 infection [4, 5]. Countries such as China and India integrate the use of traditional herbs with western medicine to boost the immune power of COVID-19 patients [6, 7]. In Indonesia itself, the use of plants as medicinal materials to meet life's needs, including maintaining health, has become one of the local wisdoms possessed by ethnic Indonesian [8]. In this context, the Indonesian government has also recommended several medicinal plants as immune boosters to prevent COVID-19 infection [9]. This study has tried to uncover the status of the use of traditional medicinal plants or herbs in Indonesia during COVID-19.

Medicinal plants are the main source of public health care in eastern Indonesia and become an integral part of their culture. The use of natural ingredients as traditional medicine in eastern Indonesia has been done for centuries, as evidenced by the existence of ancient scripts such as *Usada* and *Lontarak Pabbura* that use various plants to cure various diseases [10]. Gorontalo Province is one of the regions in Indonesia that has a unique flora wealth [11], where Gorontalo people have used various types of medicinal plants as a hereditary tradition. Traditional healers and the elderly who live in rural areas have more knowledge about traditional medicine. In addition, the limited health facilities make the surrounding population accustomed to utilising the surrounding nature in maintaining their body health. Scientific studies on the use of medicinal plants in Gorontalo Province itself are still relatively few [12, 13]. Extensive documentation is essential to reduce the loss of valuable indigenous knowledge related to plant resources in local communities. From this research, data will be obtained on the plants that are most widely used to treat coughs, colds, fever, dizziness and diarrhoea associated with mild COVID-19 symptoms and have been proven effective in the Gorontalo community. Current findings on the use of plant species drugs for the prevention of COVID-19 provide the basis for future biological efficacy testing and possible isolation of biologically active compounds to manage or prevent COVID-19.

## 2. Materials and Methods

### 2.1. Study Area

**Figure 1:**
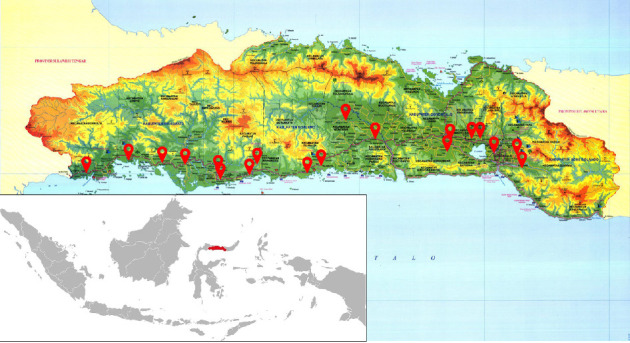
Study area, Gorontalo Province, Indonesia [14].

This research was conducted in the eastern region of Indonesia, precisely in Gorontalo Province which consists of five regencies and one city, with a total population of 1,180,948 people. The area with the most populous population is Gorontalo Regency followed by other areas, namely Bone Bolango Regency, Pohuwato Regency, Boalemo Regency and North Gorontalo Regency. Gorontalo Province is located in the northern part of Sulawesi Island, precisely at 0° 19′ 00″ to 1° 57′ 00″ north latitude and 121° 23′ 00″ to 125° 14′ 00″ east longitude of the Greenwich meridian. The location of Gorontalo Province is very strategic because it is flanked by two waters, namely Tomini Bay to the south and Sulawesi Sea to the north. The total area of Gorontalo Province is 12,435.00 km^2^, with most of the land surface being hills and having many mountains with different heights. The forest area of Gorontalo Province is 764,881.23 ha and generally consists of 25.69% conservation forest; 26.45% protected forest; 11.78% fixed production forest; 33.03% limited production forest; and conversion forest amounting to 3.04% of the total forest area of Gorontalo Province. In addition, the geographical location and elevation can be seen in Figure 1. There are 35 villages were the lowest is 3 meters above sea levels (North Buntulia Village) and the highest 692 meters above sea levels (Persatuan Village).

### 2.2. Field Interview Methods

The ethnomedicinal survey was carried out from June 2021 to March 2022. The number of samples in this study was 105 people, referring to the literature which states that for ethnographic research, including ethnomedicine, the number of respondents interviewed was 30–60 people, which was considered representative enough or reached a saturation point, where no new data were obtained [15, 16]. Ethnomedicinal data collection was carried out using field surveys, open interviews and semistructured questionnaires. Ethnobotanical information was collected through a questionnaire that had previously been tested in the existing format (the ethnobotanical survey pro forma was designed and pretested with local informants, then modified based on the informants' feedback), direct observation, discussions and interview methods [17]. The questionnaire was designed to capture information including plants used for the treatment of cough, colds, fever, dizziness and diarrhoea—several diseases related to mild COVID-19 symptoms; local name, part of the plant used, method of preparation and administration and side effects after taking the herbal ingredient. Informant biodata including age, gender, place of residence, educational and occupational background were also recorded. The informants were selected purposively using the snowball technique, where key informants (one per regency) who then recruited by other competent informants (20 per key informant) into the survey [18]. Key informants are considered to be important members of community who were observant, reflective, knew much about the culture, as well as able and willing to share their knowledge. Moreover, all informants are people who live in the study area, are over 18 years old and have been using medicinal plants to treat several diseases related to mild COVID-19 symptoms for at least the last three to five years. All informants were informed of the research concept to ask for their consent and willingness to participate in the survey. Informants who are willing to be asked individually about their knowledge regarding the use of plant species to treat several diseases related to mild COVID-19 symptoms, in this case cough, colds, fever, dizziness and diarrhoea.

### 2.3. Plant Collection and Identification

Plant species taken directly from informants were initially identified using local names and then identified by respective scientific classifications through relevant literature [19, 20]. Voucher specimens are prepared, identified and stored at the Department of Pharmacy, Faculty of Sport and Health, Universitas Negeri Gorontalo, Gorontalo, Indonesia.

### 2.4. Ethnomedicinal Data Analysis

Data analysis used descriptive and quantitative methods. The sociodemographic data of the participants were analysed using Excel software and summarised as frequencies (percentages). Ethnomedicinal data were analysed using frequency, citations and use reports (UR). UR are calculated whenever the informant cites plant species or parts used for a particular disease. UR are also calculated to determine the plant species most often used for certain diseases [21]. To determine the importance of a specific plant species by the informant, the index of relative frequency of citation (RFC) is calculated using the following equation:

(1) $$\begin{array}{c} \text{RFC}=\frac{\text{FC}}{N}, \end{array}$$

where FC (frequency of citation) is the number of mentions of certain plant species and *N* is the total number of informants in the survey [22].

To determine the preference of certain plant species for the treatment of a disease in the study area, the fidelity level (FL) is calculated using the following equation:

(2) $$\begin{array}{c} \text{FL}=\frac{I_{p}}{I_{u}}\times 100, \end{array}$$

where *I*_*p*_ is the number of informants who claim to use certain plant species in treatment and *I*_*u*_ is the total number of informants in the survey. High FL indicates the use of high plants for treatment, while low FL indicates the use of low plants for treatment [23].

In addition, to identify community agreement regarding the types of plants used to treat certain diseases, the informant consensus factor (ICF) through equation (3) can be used as follows [24]:

(3) $$\begin{array}{c} \text{ICF}=\frac{\left(N_{ur}-N_{t}\right)}{\left(N_{ur}-1\right)}, \end{array}$$

where *N*_*ur*_ is the number of uses of plant species for each disease category and *N*_*t*_ is the number of species used for each particular category by all respondents.

## 3. Results and Discussion

The following are the results of our research from the participants were individually questioned on their knowledge regarding the use plant species to treat several diseases related to mild COVID-19 symptoms such as cough, colds, fever, dizziness and diarrhoea. Five native field assistants were engaged to administer and interpret the questions to the participants in their local language (Gorontalo) in order to overcome the language barrier and to facilitate efficient communication.

### 3.1. The Demographic Characteristics of the Informants

**Figure 2:**
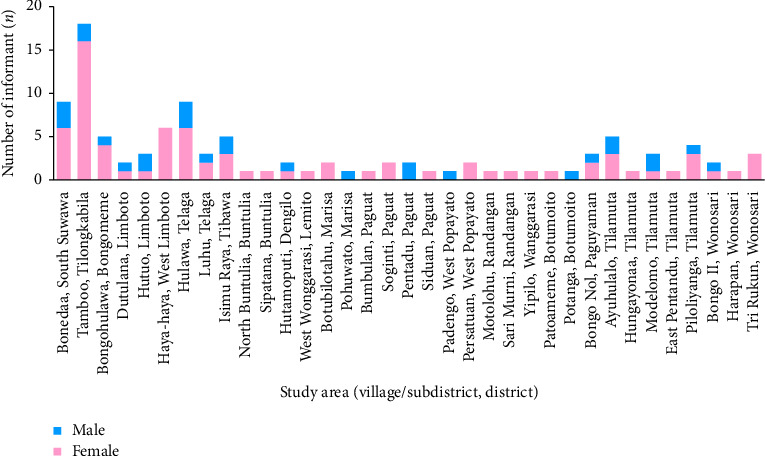
Studied villages in the Gorontalo Province with in-detail demographic characteristics of the local informants (*n* = 105).

A total of 105 local informants (28 males and 77 females) aged between 21 and 80 years were interviewed from 35 villages (see Figure 2). Demographic characteristics including gender, age group, education level and occupation are recorded (see Table 1).

### 3.2. Botanical Diversity

**Figure 3:**
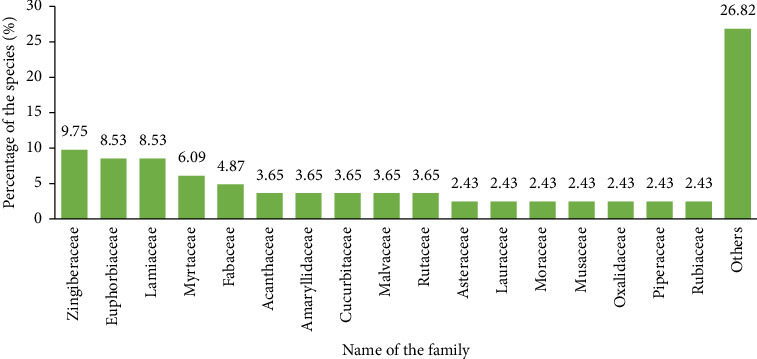
Families of plants with the percentage of their species for managing several diseases related to mild COVID-19 symptoms in Gorontalo Province, Indonesia.

In the current study, 82 locally used species of medicinal plants from 40 families were recorded for the treatment and management of several diseases related to mild COVID-19 symptoms such as cough, colds, fever, dizziness and diarrhoea in the study area (see Table 2). Zingiberaceae, Euphorbiaceae and Lamiaceae are the three main families most representative for the 82 species documented as remedies against cough, colds, fever, dizziness and diarrhoea. Zingiberaceae have eight species, while Euphorbiaceae and Lamiaceae each have seven species (see Figure 3) and the other 37 families each have one representative species.

Information on local names, drug use, plant parts used, preparation and administration methods is also shown in Table 2. Based on UR data, the most widely used species to overcome cough are *Plectranthus scutellarioides* (41 UR), followed by *Zingiber officinale* (24 UR), *Curcuma longa* (19 UR) and *Citrus aurantiifolia* (10 UR). Another ethnomedicine study revealed that *P. scutellarioides* was used as a potent cough medicine by traditional medicine in the eastern highlands' region of Papua New Guinea [25]. In vitro studies revealed that *P. scutellarioides* leaf extract was shown to have antibacterial activity against test bacteria that cause cough such as *Streptococcus pneumonia*, *Klebsiella pneumonia*, *Staphylococcus aureus*, *Staphylococcus epidermidis*, *Enterobacter agglomerans* and *Candida albicans* fungi, with a minimum inhibitory concentration (MIC) of 0.1%–0.75% w/v, and a minimum killing concentration (MKC) of 0.25%–1.75% w/v [26]. The extract also has the potential as a sputum diluent based on a decrease in mucus viscosity with a concentration of 0.01%–0.1% w/v. The effective dose of *P. scutellarioides* leaf extract that can be used as a reference to cure sputum is 1.75% w/v which is equivalent to seven fresh *P. scutellarioides* leaves [26].

To overcome colds, *Z. officinale* (19 UR) are most widely used, followed by *Ocimum basilicum* (17 UR). *In silico* studies with molecular docking revealed that the active compound allicin present in *Z. officinale* has anti-influenza cytokines [27]. In vitro studies revealed that crude extract mixtures of *Z. officinale*, honey and garlic inhibited influenza A virus growth in human peripheral blood mononuclear cells cultured [28]. *Z. officinale* ethanol extract has also been reported to inhibit the growth and development of H5N1 virus in a dose-dependent manner [29]. In vivo studies have revealed that treatment with gingerenone A, a compound isolated from *Z. officinale*, can suppress the replication of influenza A virus in the lungs of H5N1 virus-infected mice, reduce body weight loss and prolong their survival [30].

To overcome fever, a widely used species is *Bryophyllum pinnatum* (15 UR), followed by *C. longa* (15 UR), *Jatropha curcas* (14 UR) and *Z. officinale* (12 UR). *B. pinnatum* leaf methanol extract has been studied for anti-inflammatory, analgesic and antipyretic activity in animal models. The extract resulted in significant inhibition of carrageenan-induced paw oedema, significant reduction in cotton pellet granuloma in rats, inhibition of acetic acid-induced writhing in rats and significant dose-dependent reduction of yeast-induced fever [31]. *Allium sativum* (17 UR) and *Z. officinale* (10 UR) are widely used to deal with dizziness. A case study report in Australia revealed that administration of 1.2 g aged garlic extract (AGE) as a daily food supplement intervention was able to reduce the frequency and severity of headaches experienced by patients with suboptimal management of episodic tension headache after 6 weeks of treatment [32]. The effect is thought to be derived from the powerful anti-inflammatory effect of AGE on microvasculature [33]. AGE, a stable homogeneous extract rich in S-allyl-cysteine, may increase microvascular dilatation as a consequence of increased or imitation of prostaglandin vasodilatation [34].

Meanwhile, *Psidium guajava* (69 UR), both leaves and fruits, became the most widely used species to overcome diarrhoea, followed by *Myristica fragrans* (20 UR) and *C. longa* (13 UR). In vitro studies revealed that *P. guajava* has antibacterial activity against diarrhoea-causing bacteria such as *Escherichia coli*, *Salmonella* sp., *Shigella* sp., *Staphylococcus aureus* and *Vibrio cholerae* [35–37], and antivirals against rotaviruses causing diarrhoea in simians [38]. Spasmolytic activity of *P. guajava* against ileum isolated from guinea pig has also been reported [39–41]. The administration of *P. guajava* leaf extract in various animal models of diarrhoea such as mice [42, 43], rats [44–46] and rabbits [47] showed significant antidiarrheal activity. Quercetin as the main biomarker in *P. guajava* contributes to antidiarrheal activity through the mechanism of inhibition of intestinal secretion, reduction of nitrate oxide production and inflammatory expression and reactivation of Na^+^/K^+^-ATPase activity [48], as well as inhibition of SepA protease activity, a protein produced by *S. flexneri* [49]. Clinical studies revealed that the administration of *P. guajava* in 62 patients with rotavirus enteritis had a good curative effect with a recovery rate of 87.1% and a shorter diarrhoea discontinuation time (25.1 ± 9.5 h) compared to the control [50]. In addition, a randomised, double-blind clinical study of 50 patients with acute diarrhoea who received the product *P. guajava* leaf capsule (500 mg) orally every 8 h for 3 days reported that the product could reduce the duration of abdominal pain in patients [51]. In addition, we have summarised the reported bioactivities and isolated compounds or extracts that are relevant to the use of plant species with high reported use for treating coughs, colds, fever, dizziness and diarrhoea (see Table 3).

### 3.3. Ranking of the Most Important Medicines

Although the number of plant species used for the treatment and care of several diseases related to mild COVID-19 symptoms is relatively high, their ethnobotanical indices include RFC (0.01–0.41) and FL (1%–41%) generally low (see Table 2). Furthermore, several diseases related to mild COVID-19 symptoms (where the informants offered treatment and declared themselves cured) were divided into five disease categories, and the informants' level of agreement with their treatment was evaluated. The categories of diarrhoea (ICF 0.85), cough (ICF 0.78), colds (ICF 0.73), dizziness (0.72) and fever (ICF 0.71) showed relatively stronger agreement between the informants (see Table 4).

The results show that diarrhoea has the highest ICF value, which indicates that the plant is widely known and often used in society for certain health problems. In addition, there is significant cultural importance associated with the use of this plant, which encourages collective understanding of its benefits. These findings correlate with other studies reported by Bhagawan et al. [68] among the Tengger Tribe Community who live in Argosari Village, East Java, Indonesia. A high ICF value reflects the popularity and effectiveness of this plant-based medicine among the informants. This shows that the local community really appreciates the medicinal properties and various uses of this plant.

However, current research reveals that *P. guajava* is the most popular plant species with a high index value (RFC 0.62, FL 62%), which can be taken as a signal of the excellent curative potential of the plant. Khan et al. [69] revealed that plants used repeatedly were more phytochemically active. Quercetin, which is the most dominant flavonoid in *P. guajava*, shows strong antidiarrheal activity [70]. The antidiarrheal activity of quercetin is thought to be derived from the relaxing effect on the intestinal muscle layer that prevents intestinal contraction [71].

### 3.4. Plant Parts Used

**Figure 4:**
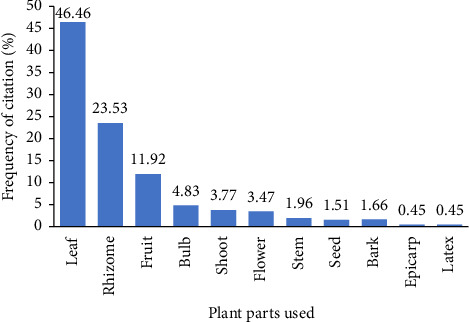
Plant parts used for managing several diseases related to mild COVID-19 symptoms in Gorontalo Province, Indonesia.

Diverse plant parts such as leaves, rhizome, fruit, bulb, shoot, flower, stem, seed, bark, epicarp and latex are used to overcome several diseases related to mild COVID-19 symptoms that have been reported by informants (see Figure 4). However, leaves (46.46%) were the main plant used to overcome several diseases related to mild COVID-19 symptoms, followed by rhizome (23.53%) and fruit (11.92%).

These results show the widespread use of leaves as the main part of plants in the manufacture of traditional medicines to treat human health problems. In particular, this study determined that leaves are the part of the plant most widely used to treat human diseases in the region, a finding that is consistent with various other studies conducted in various regions in Indonesia [72]. There are several reasons that can explain the dominance of leaves in traditional drug formulations for human health. As pointed out by Bekele et al. [73], leaves are often preferred because of their relative ease of preparation and the potential therapeutic benefits they offer compared to other plant parts. Leaves are generally easier to obtain, easier to harvest and may contain higher concentrations of active medicinal compounds, making them a practical choice for the manufacture of traditional medicines. Overall, these insights can inform the development of sustainable harvesting practices and conservation strategies to ensure the continued availability of this valuable natural resource.

### 3.5. Preparation and Application Modes

**Figure 5:**
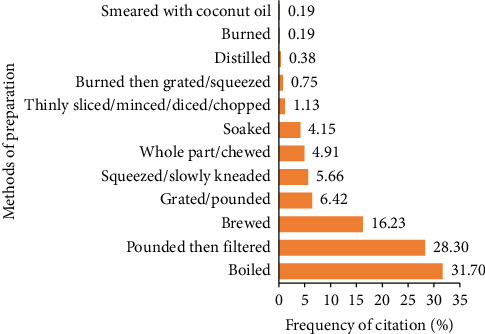
Methods of preparing plant species used for managing several diseases related to mild COVID-19 symptoms in Gorontalo Province, Indonesia.

To prepare medicinal plants to overcome several diseases related to mild COVID-19 symptoms, the informants identified 12 methods in which most (about 31.70%) were prepared by boiling (see Figure 5). These results show consistency with the findings of previous studies [74], which was carried out in the western region of Indonesia, namely Java and Bali. According to Kamatenesi et al. [75], boiling helps extract active ingredients from parts of medicinal plants which can increase the efficacy of medicines against various diseases, as well as preserving herbal medicines longer than using cold extraction. However, in some cases, boiling can cause degradation of bioactive materials, especially aromatic compounds, if it takes a long time [76]. This highlights the importance of investigating the effectiveness of the medicinal plant preparation methods used, because the active ingredients of medicinal plants can vary based on the extraction method used. In addition, medicinal plant species are also managed individually by pounding and filtering (28.30%) or brewing (16.23%).

**Figure 6:**
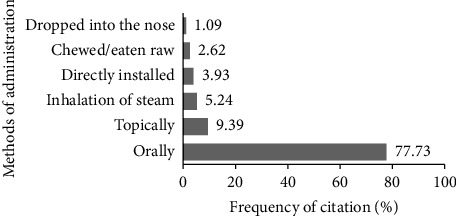
Methods of administering plant species used for managing several diseases related to mild COVID-19 symptoms in Gorontalo Province, Indonesia.

The most common preparation method, boiling, influences the administration method of this medicinal plant. In terms of administration, almost all preparations were administered orally (77.73%) by informants in the study area (see Figure 6). These results are in accordance with [77] which was reported in Pacitan Regency, East Java, Indonesia. Kassa et al. [78] reported that oral administration offers unique advantages to practitioners of traditional medicine, allowing them to prevent complications during antidote treatment. In some cases, such as fever, whole fresh plant parts or preparations that have been pounded and then filtered (*tumbuk serkai*) are attached directly or compressed to the patient's forehead. Preparations that have been boiled or brewed for inhalation of steam are given to patients who experience colds or dizziness, while diarrhoea patients generally consume *P. guajava* fruit directly.

### 3.6. Toxicity and Side Effects of Herbal Remedies

Traditional medicine, like other forms of treatment, also has potential side effects, although relatively small, and in the current study can be seen in Table 5. Results like this were also reported in research conducted in Artuma Fursi District, Amhara State, Ethiopia [79]. The existence of data on undesirable effects in traditional use is very valuable information for the clinical use of these plants in terms of safety.

The informants reported several plants such as *Artocarpus altilis*, *Annona muricata*, *Ocimum basilicum*, *Muntingia calabura* and *Piper betle* can cause a drowsiness effect after consuming it to treat certain diseases. However, the resulting drowsiness effect could occur due to other pharmacological activities of the plant. For example, *O. basilicum* has been reported to improve sleep quality and overcome insomnia in menopausal women [80].

Undesirable effects can also be caused by the use of medicinal plants that exceed the treatment dose so that they can cause side effects that are the opposite of the aim of the treatment. For example, consumption of *P. guajava* exceeding the dose can actually cause constipation in patients with diarrhoea [51]. This brings special attention to the importance of determining the appropriate dosage of medication and investigating the safety of the use of these medicinal plants.

All data regarding side effects were recorded based on quotes from participants, which were then analysed qualitatively. In the context of this study, ‘side effects' refers to negative reactions or undesirable effects that may occur when consuming herbal treatments, ranging from mild symptoms such as stomach aches to more serious issues like allergic reactions, depending on the herbal formulation and individual sensitivity; essentially, these are unintended consequences of consuming herbal supplements, similar to how side effects are described in conventional medicine.

Generally, the side effects reported by participants occurred after consuming the herbal preparations they made to address several diseases related to mild COVID-19 symptoms. No further reports have been made regarding the side effects of long-term consumption. To clarify the accuracy of the reported side effects, further evaluation is necessary.

## 4. Conclusions

Ethnomedicinal studies that have been carried out reveal the use of plant species and the wealth of knowledge of key informants, and we documented the use of 82 plant species for the treatment of several diseases related to mild COVID-19 symptoms, such as cough, fever, colds, dizziness and diarrhoea, in Gorontalo Province, eastern Indonesia. This study is important because it contributes to the preservation of the traditional medicinal knowledge of local residents in Gorontalo Province, Eastern Indonesia, which is increasingly being eroded. Our findings indicate that *Plectranthus scutellarioides* (L.) R.Br. is predominantly used to cope with cough, *Zingiber officinale* Roscoe to cope with colds, *Bryophyllum pinnatum* (Lam.) Oken to cope with fever, *Allium sativum* L. to cope with dizziness and *Psidium guajava* L. to cope with diarrhoea. Overall, current research reveals that *P. guajava* is the most popular plant species with a high index value (RFC 0.62, FL 62%), which can be taken as a signal of the excellent curative potential of the plant. It is highly recommended that plants obtained from ethnomedicinal studies undergo scientific validation of the active compounds, claims for efficacy and safety of these plants because adequate standardisation, quality control and safety aspects will greatly support the use of the above plant extracts for clinical use.

## Figures and Tables

**Table 1:** Demographic characteristics of the informants (*n* = 105) in the study area.

Characteristics	Group	No. of informants, *n* (%)
Gender	Male	28 (26.67)
	Female	77 (73.33)

Age range (years)	17–25	2 (1.90)
	26–35	4 (3.81)
	36–45	20 (19.05)
	46–55	49 (46.67)
	56–65	23 (21.90)
	≥ 66	7 (6.67)

Education level	Primary	35 (33.33)
	Secondary	39 (37.14)
	Tertiary (high school)	28 (26.67)
	Undergraduate	3 (2.86)

Occupation	Traditional healer	6 (5.71)
	Housewife	65 (61.90)
	Farmer	17 (16.19)
	Entrepreneur	9 (8.57)
	Employee	5 (4.76)
	Retired	3 (2.86)

**Table 2:** Diversity of plant species used for treating several diseases related to mild COVID-19 symptoms in Gorontalo Province, Indonesia.

No.	Botanical name [family]	Voucher no.	Vernacular name	Ailments (UR)	RFC	FL (%)	Part used	Preparation	Application
1.	*Andrographis paniculata* (Burm.f.) Nees [Acanthaceae]	GOR-2162	*Sambilote*	C (1); F (7)	0.08	8	L	Boiled then mixed with honey; or pounded then filtered	Orally

2.	*Hemigraphis colorata* W.Bull [Acanthaceae]	GOR-2104	*Lelenggota*	F (1)	0.01	1	L	Squeezed; or pounded then filtered	Orally

3.	*Strobilanthes crispa* Blume [Acanthaceae]	GOR-2172	*Kecik beling*	D (1)	0.01	1	L	Boiled	Orally

4.	*Amaranthus tricolour* L. [Amaranthaceae]	GOR-2173	*Bayam merah*	DZ (1)	0.01	1	L	Boiled	Orally

5.	*Allium cepa* L. [Amaryllidaceae]	GOR-2110	*Bawang merah*	F (7); DZ (3)	0.10	10	Bu	Thinly sliced; boiled; or pounded then filtered	Directly installed or compressed; topically; or orally

6.	*Allium sativum* L. [Amaryllidaceae]	GOR-2111	*Bawang putih*	CC (4); DZ (17)	0.20	20	Bu	Fresh whole bulb or boiled; brewed; soaked; or pounded then filtered	Inhalation of steam; orally; or topically

7.	*Allium tuberosum* Rottler ex Spreng. [Amaryllidaceae]	GOR-2109	*Kucai*	F (10)	0.10	10	L	Boiled; pounded then filtered; or cut into small pieces then mixed with oil	Orally; or topically

8.	*Mangifera indica* L. [Anacardiaceae]	GOR-2106	*Mangga*	F (1)	0.01	1	Ba	Boiled	Orally

9.	*Annona muricata* L. [Annonaceae]	GOR-2167	*Nangka Belanda*	C (1); DZ (3)	0.04	4	L	Boiled; or pounded then filtered	Orally

10.	*Centella asiatica* (L.) Urb. [Apiaceae]	GOR-2155	*Tingalahula*	C (3); F (1); DZ (1)	0.05	5	L	Boiled; or pounded then filtered	Orally

11.	*Alstonia scholaris* (L.) R. Br. [Apocynaceae]	GOR-2158	*Talanggilala*	C (1)	0.01	1	Ba	Boiled; brewed; or soaked	Orally

12.	*Blumea balsamifera* (L.) DC. [Asteraceae]	GOR-2163	*Tapulapunga*	C (2); CC (1); F (1)	0.04	4	L; Sh	Boiled; or pounded then filtered	Orally

13.	*Vernonia amygdalina* Delile [Asteraceae]	GOR-2174	*Aprika*	F (1)	0.01	1	L	Boiled	Orally

14.	*Anredera cordifolia* (Ten.) Steenis [Basellaceae]	GOR-2115	*Binahong*	C (1); CC (1)	0.02	2	L	Boiled	Orally

15.	*Carica papaya* L. [Caricaceae]	GOR-2156	*Daun papaya*	F (8)	0.08	8	L	Soaked then mixed with brown sugar; boiled; brewed; or pounded then filtered	Orally

16.	*Garcinia* x *mangostana* L. [Clusiaceae]	GOR-2147	*Manggis*	C (1); CC (1); F (1); D (3)	0.04	4	L; Ep	Boiled; or pounded then filtered	Orally

17.	*Commelina diffusa* Burm.f. [Commelinaceae]	GOR-2159	*Hulotua*	C (6); F (8)	0.13	13	L	Boiled; or pounded then filtered	Orally

18.	*Bryophyllum pinnatum* (Lam.) Oken [Crassulaceae]	GOR-2119	*Cocor bebek*	F (15)	0.14	14	L	Boiled; thinly sliced or squeezed; or pounded then filtered	Orally; or directly installed or compressed

19.	*Lagenaria siceraria* (Molina) Standl. [Cucurbitaceae]	GOR-2143	*Sayur walu*	F (1)	0.01	1	Fr	Squeezed	Orally

20.	*Momordica charantia* L. [Cucurbitaceae]	GOR-2153	*Beleng gede*	C (1); CC (1)	0.02	2	L	Boiled; or pounded then filtered	Orally

21.	*Momordica cochinchinensis* (Lour.) Spreng. [Cucurbitaceae]	GOR-2123	*Dumbaya*	C (1)	0.01	1	L	Brewed	Orally

22.	*Acalypha indica* L. [Euphorbiaceae]	GOR-2175	*Akar kucing*	C (1)	0.01	1	R	Pounded then filtered	Orally

23.	*Codiaeum variegatum* (L.) Rumph. ex A.Juss. [Euphorbiaceae]	GOR-2176	*Puring merah*	D (1)	0.01	1	L	Boiled; or pounded then filtered	Orally

24.	*Euphorbia hirta* L. [Euphorbiaceae]	GOR-2154	*Tabulotutu*	C (1); F (1)	0.01	1	L	Pounded then filtered	Orally

25.	*Jatropha curcas* L. [Euphorbiaceae]	GOR-2129	*Balacae, Bindalo*	C (4); CC (2); F (14); DZ (5); D (9)	0.30	30	L, Lx	Boiled; brewed; or pounded then filtered	Orally; or compressed

26.	*Jatropha gossypiifolia* L. [Euphorbiaceae]	GOR-2177	*Balacae merah*	DZ (1)	0.01	1	L	Boiled	Orally

27.	*Manihot esculenta* Crantz [Euphorbiaceae]	GOR-2165	*Dungo ubi*	DZ (1); D (1)	0.02	2	L	Boiled; or wet with water then scraped or grated	Orally; or topically

28.	*Melanolepis multiglandulosa* (Reinw. ex blume) Rchb. and Zoll. [Euphorbiaceae]	GOR-2121	*Walongo*	C (1); DZ (1)	0.02	2	L, St	Squeezed; or scraped	Orally

29.	*Erythrina subumbrans* (Hassk.) Merr. [Fabaceae]	GOR-2178	*Polabu*	F (1)	0.01	1	L	Fresh whole leaves	Directly installed or compressed

30.	*Senna tora* (L.) Roxb. [Fabaceae]	GOR-2132	*Kaca lo udu*	D (2)	0.02	2	L	Boiled	Orally

31.	*Sesbania grandiflora* (L.) Pers. [Fabaceae]	GOR-2170	*Turi*	C (1); CC (1); F (1); DZ (8); D (1)	0.10	10	L, St	Boiled; brewed; or pounded then filtered	Orally; inhalation of steam; or topically

32.	*Tamarindus indica* L. [Fabaceae]	GOR-2108	*Asang jawi*	C (1); CC (1); F (1)	0.01	1	Fr	Brewed	Orally

33.	*Clerodendrum japonicum* (Thunb.) Sweet [Lamiaceae]	GOR-2117	*Sesebanua*	F (1)	0.01	1	L	Fresh whole leaves	Directly installed or compressed

34.	*Plectranthus amboinicus* (Lour.) Spreng. [Lamiaceae]	GOR-2120	*Daun tebal*	C (3); F (1)	0.04	4	L	Boiled; or pounded then filtered	Orally

35.	*Plectranthus scutellarioides* (L.) R.Br. [Lamiaceae]	GOR-2150	*Mayana*	C (41); CC (3); F (4)	0.41	41	L	Boiled; brewed; soaked; pounded then filtered; or distilled	Orally; or inhalation of steam

36.	*Ocimum americanum* L. [Lamiaceae]	GOR-2135	*Kemangi*	F (1); DZ (1)	0.01	1	L	Boiled; or pounded then filtered	Orally; or compressed

37.	*Ocimum basilicum* L. [Lamiaceae]	GOR-2136	*Ulu-ulu*	CC (17); F (1)	0.17	17	L	Boiled; soaked; or pounded then filtered	Inhalation of steam; dropped into the nose; orally; or topically

38.	*Ocimum tenuiflorum* L [Lamiaceae]	GOR-2137	*Kemangi merah*	CC (3)	0.03	3	L	Brewed; or pounded then filtered	Inhalation of steam; or orally

39.	*Orthosiphon aristatus* (Blume) Miq. [Lamiaceae]	GOR-2140	*Kumis kucing*	C (6); F (3)	0.07	7	L	Boiled; or pounded then filtered	Orally; or topically

40.	*Cinnamomum verum* (J. Presl) [Lauraceae]	GOR-2133	*Kayu manis*	C (2); DZ (5)	0.07	7	Ba	Boiled; chewed; or pounded then filtered	Orally; or topically

41.	*Persea americana* Mill. [Lauraceae]	GOR-2107	*Alpukat*	DZ (1)	0.01	1	L	Boiled	Orally

42.	*Abelmoschus manihot* (L.) Medik. [Malvaceae]	GOR-2124	*Daun gedi*	C (1); F (1)	0.02	2	L	Boiled; or brewed	Orally

43.	*Hibiscus mutabilis* L. [Malvaceae]	GOR-2171	*Molowahu*	F (1)	0.01	1	Sh	Pounded then filtered	Orally

44.	*Hibiscus rosa-sinensis* L. [Malvaceae]	GOR-2179	*Ulange*	F (1)	0.01	1	L	Pounded then filtered	Topically

45.	*Aglaia odorata* Lour. [Meliaceae]	GOR-2101	*Pacar cina*	C (1)	0.01	1	L	Boiled	Orally

46.	*Tinospora crispa* (L.) Hook. f. and Thomson [Menispermaceae]	GOR-2116	*Tali pahit*	F (3)	0.03	3	St	Brewed; or pounded then filtered	Orally

47.	*Artocarpus altilis* (Parkinson ex F.A.Zorn) Fosberg [Moraceae]	GOR-2168	*Daun amo*	C (4); CC (2)	0.05	5	L	Boiled; brewed; soaked; or burned	Orally; or inhalation of steam

48.	*Artocarpus heterophyllus* Lam. [Moraceae]	GOR-2151	*Daun nangka*	DZ (3)	0.03	3	L	Boiled	Orally; or topically

49.	*Moringa oleifera* Lam. [Moringaceae]	GOR-2134	*Kelor*	F (1); DZ (3)	0.04	4	L	Boiled; brewed; or soaked	Orally

50.	*Muntingia calabura* L. [Muntingiaceae]	GOR-2139	*Kersen*	C (1); F (1); DZ (5); D (1)	0.08	8	L, Fr	Boiled; or fresh whole fruits	Orally; or eaten raw

51.	*Musa acuminata* [Musaceae]	GOR-2180	*Pisang sepatu*	F (1)	0.01	1	Lx	Brewed	Orally

52.	*Musa acuminata* ‘lady finger' [Musaceae]	GOR-2157	*Lambi lo susu*	D (2)	0.02	2	Fr	Fresh whole fruit	Eaten raw

53.	*Myristica fragrans* Houtt. [Myristicaceae]	GOR-2152	*Pala*	CC (1); F (1); DZ (1); D (20)	0.20	20	Fr, S	Burned then grated and mixed with water; or pounded then filtered	Orally; or topically

54.	*Melaleuca leucadendra* (L.) L. [Myrtaceae]	GOR-2103	*Kayu putih*	CC (2); DZ (1)	0.03	3	L	Pounded then filtered; or distilled	Topically (apply on the back or forehead); or inhalation of steam

55.	*Psidium guajava* L. [Myrtaceae]	GOR-2128	*Jambu biji*	D (69)	0.62	62	Fr	Fresh whole fruits	Eaten raw
							L	Boiled; or pounded then filtered	Orally

56.	*Syzygium aromaticum* (L.) Merr. and L.M.Perry [Myrtaceae]	GOR-2118	*Hungolawa*	C (1); CC (2); F (1); DZ (3); D (3)	0.08	8	Fr, Fl	Boiled; or brewed; or pounded then filtered	Inhalation of steam; orally; or topically

57.	*Syzygium cumini* (L.) Skeels [Myrtaceae]	GOR-2127	*Daun jambolang*	D (2)	0.02	2	L	Brewed	Orally

58.	*Syzygium polyanthum* (Wight) Walp. [Myrtaceae]	GOR-2161	*Salam*	DZ (1)	0.01	1	L	Boiled; or pounded then filtered	Orally

59.	*Averrhoa bilimbi* L. [Oxalidaceae]	GOR-2113	*Lembetue*	C (9); CC (7)	0.13	13	Fl, Fr, L	Fresh whole flowers or boiled; brewed; distilled; soaked; or pounded then filtered	Inhalation of steam; dropped into the nose; or orally

60.	*Averrhoa carambola* L. [Oxalidaceae]	GOR-2112	*Belimbing manis*	CC (1)	0.01	1	L, Fr	Pounded then filtered	Orally

61.	*Phyllanthus niruri* L. [Phyllanthaceae]	GOR-2149	*Duku ana*	C (1); D (1)	0.02	2	L	Boiled; or brewed	Orally

62.	*Piper betle* L. [Piperaceae]	GOR-2166	*Sirih*	C (3); CC (8); F (2); DZ (1)	0.10	10	L, Fl	Fresh whole leaves or boiled; soaked then mixed with salt; or pounded then filtered	Inhalation of steam; compressed; or orally

63.	*Piper nigrum* L. [Piperaceae]	GOR-2144	*Rica Jawa*	C (1); CC (1); D (1)	0.03	3	Fr	Brewed; chewed; or boiled	Orally

64.	*Cymbopogon citratus* (DC.) Stapf [Poaceae]	GOR-2164	*Timbuwale*	C (4); CC (3); F (3); DZ (3)	0.12	12	St, L, Fr	Boiled; brewed; soaked; or pounded then filtered	Inhalation of steam; or orally

65.	*Labisia pumila* (Blume) Fern.-Vill. [Primulaceae]	GOR-2160	*Rumput fatimah*	D (1)	0.01	1	L	Boiled	Orally

66.	*Ziziphus spina-christi* (L.) Desf. [Rhamnaceae]	GOR-2114	*Bidara*	D (1)	0.01	1	L	Boiled	Orally

67.	*Morinda citrifolia* L. [Rubiaceae]	GOR-2148	*Menggudu*	C (1); CC (3)	0.04	4	Fr	Boiled; or brewed	Orally

68.	*Uncaria gambir* (Hunter) Roxb. [Rubiaceae]	GOR-2102	*Gambir*	D (1)	0.01	1	Fr	Soaked	Orally

69.	*Citrus aurantifolia aurantiifolia* (Christm.) Swingle [Rutaceae]	GOR-2131	*Daun lemon*	C (10); CC (2); F (2); DZ (7)	0.16	16	Fr, L	Boiled; brewed; grated; soaked; squeezed; fresh whole leaves or pounded then filtered	Inhalation of steam; or orally

70.	*Citrus limon* (L.) Osbeck [Rutaceae]	GOR-2105	*Lemon suanggi*	C (7); CC (3); F (1); DZ (7)	0.17	17	Fr	Brewed; or squeezed then mixed with soy sauce or whiting	Orally; or topically

71.	*Citrus sinensis* (L.) Osbeck [Rutaceae]	GOR-2130	*Jeruk peras*	F (1)	0.01	1	Fr	Boiled	Topically

72.	*Camellia sinensis* (L.) Kuntze [Theaceae]	GOR-2122	*Daun teh*	D (1)	0.01	1	L	Brewed and then mixed with salt to taste	Orally

73.	*Phaleria macrocarpa* (Scheff.) Boerl. [Thymelaeaceae]	GOR-2146	*Mahkota dewa*	DZ (1)	0.01	1	Fr	Boiled	Orally

74.	*Lantana camara* L. [Verbenaceae]	GOR-2181	*Bituke*	DZ (1)	0.01	1	L, Fl	Boiled; brewed; soaked; or pounded then filtered	Orally

75.	*Alpinia galanga* (L.) Willd. [Zingiberaceae]	GOR-2145	*Linggoboto*	CC (1)	0.01	1	Rh	Boiled	Orally

76.	*Curcuma longa* L. [Zingiberaceae]	GOR-2141	*Alawahu*	C (19); CC (6); F (15); DZ (4); D (13)	0.37	37	Rh	Boiled; brewed; soaked; or pounded then filtered and mixed with honey or sugar to taste	Orally; or topically

77.	*Curcuma zanthorrhiza* Roxb. [Zingiberaceae]	GOR-2169	*Temulawak*	C (1); CC (1); F (3); DZ (2); D (1)	0.07	7	Rh	Boiled; or pounded then filtered	Orally; or compressed

78.	*Curcuma zedoaria* (Christm.) Roscoe [Zingiberaceae]	GOR-2142	*Kunyit putih*	CC (1)	0.01	1	Rh	Brewed	Orally

79.	*Kaempferia galanga* L. [Zingiberaceae]	GOR-2138	*Humopoto*	C (6); CC (8); F (2); DZ (3)	0.12	12	Rh	Boiled; brewed; grated; or pounded then filtered	Inhalation of steam; or orally

80.	*Zingiber montanum* (J.Koenig) Link ex A.Dietr. [Zingiberaceae]	GOR-2182	*Bungale*	C (1)	0.01	1	Rh	Boiled	Orally

81.	*Zingiber officinale* Roscoe [Zingiberaceae]	GOR-2125	*Goraka, Melito*	C (24); CC (19); F (12); DZ (10); D (2)	0.40	40	Rh	Boiled; brewed; soaked; or pounded then filtered	Inhalation of steam; or orally

82.	*Zingiber officinale* var. *rubrum* [Zingiberaceae]	GOR-2126	*Jahe merah*	C (2); F (1)	0.02	2	Rh	Boiled; or brewed	Orally

*Note:* Ailments (C: cough, CC: colds, F: fever, DZ: dizziness, and D: diarrhoea); part used (Ba: bark, Bu: bulb, Ep: epicarp, Fl: flower, Fr: fruit, Lx: latex, L: leaf, R: root, Rh: rhizome, S: seed, Sh: shoot, and St: stem).

Abbreviations: FL = fidelity level, RFC = relative frequency of citation, and UR = use reports.

**Table 3:** Summary of reported bioactivities and isolated compounds or extracts relevant to the use of plant species with high reported uses.

Ailments	Species (UR)	Secondary metabolites	Bioactivity	Ref.
Cough	*Plectranthus scutellarioides* (41)	Trans 13-octadecenoic acid; flavonoids; tannins; polyphenols; saponins	*P. scutellarioides* can kill the bacteria (*Streptococcus pneumoniae*, *Klebsiella pneumoniae*, *Staphylococcus epidermidis*, *Staphylococcus aureus* and *Enterobacter agglomerans*) which is the cause of the common manifestation of cough symptoms	[26, 52]
	*Zingiber officinale* (24)	Gingerol; phenol; flavonoid; terpenoid; essential oils	*Z. officinale* extract with a dose of 25 mg/kg body weight can inhibit the cough reflexes and reduce the number of coughs in guinea pigs	[53, 54]
	*Curcuma longa* (19)	Curcuminoid (curcumin); essential oils	Curcumin contained in *C. longa* rhizomes often used to treat coughs, sore throats and respiratory disorders as a home remedy. Apart from increasing the body's immunity, *C. longa* rhizomes can inhibit important proteins of SARS-CoV-2, so it can be used as a therapeutic or protective drug against SARS-CoV-2	[55]
	*Citrus aurantiifolia* (10)	Propanedioic acid, dimethyl ester; saponin; alkaloid; flavonoid; tannin	*C. aurantiifolia* has greater activity against Gram-negative bacteria (*Klebsiella pneumoniae*) compared to Gram-positive bacteria (*Staphylococcus aureus* and *Staphylococcus* ssp.) isolated from patient sputum sample	[56, 57]

Colds	*Zingiber officinale* (19)	Gingerol; phenol; flavonoid; terpenoid; essential oils	*Z. officinale* can stimulate mucosal cells to secrete IFN-β which is responsible for preventing viral infections by reducing the attachment and internalisation of viruses so that they can relieve cold symptoms	[53, 58]
	*Ocimum basilicum* (17)	Apigenin-7-glucuronide; dihydrokaempferol-3-glucoside	*O. basilicum* against SARS-CoV-2 in an in silico test showed that the polyphenolic constituents apigenin-7-glucuronide and dihydrokaempferol-3-glucoside had binding affinities of −8.77 Kcal/mol and −8.96 Kcal/mol, respectively, which had great potential as antiviral activity. These compounds have binding affinity with the main protease enzyme in SARS-CoV-2	[59]

Fever	*Bryophyllum pinnatum* (15)	Bryotoxins; daigremontianin; bryophyllins; kalantubosides; bufadienolides; kaempferol; luteolin; phenolic acid; flavonoid	Anti-inflammatory effect of *B. pinnatum* may be due to the effects on human neutrophils and their ability to suppress the expression of macrophage migration inhibitory factors. Neutrophils releases cytokines including IL1, IL-6, TNF-α, interferon *γ* and others. These proinflammatory cytokines in turn encourage the liver to synthesise various acute-phase reactant proteins and also produce systemic inflammatory responses such as fever and leucocytosis	[60, 61]
	*Curcuma longa* (15)	Flavonoid; anthraquinone	Phenolic compounds, namely flavonoids and anthraquinones in *C. longa*, can inhibit lipid peroxidation and increase the antioxidant effect in counteracting free radicals caused by *Salmonella* and oxidative stress in typhoid fever	[62]
	*Jatropha curcas* (14)	Tannin; alkaloid; saponin; flavonoids; terpenoid; glycosides; steroid	At a dose of 250 mg/kg body weight, *J. curcas* extract has effectiveness as an antipyretic. The active components of the plant, namely flavonoids, can inhibit prostaglandins, protein kinases, monoamine oxidase, DNA polymerase and cyclooxygenase which trigger fever	[63]

Dizziness	*Allium sativum* (17)	Ajoene; allicin; allyl methyl thiosulfinate; methyl allyl thiosulfinate	Activity of *A. sativum* extract demonstrated protective activity against influenza viruses by increasing the production of neutralising antibodies when administered to mice	[64]
	*Zingiber officinale* (10)	Gingerol; phenol; flavonoid; terpenoid; essential oils	*Z. officinale* extract at an oral dose of 1 g proven effective in clinical studies of induced motion sickness and demonstrated superiority in vertigo reduction	[53, 65]

Diarrhoea	*Psidium guajava* (69)	Guajaphenone A; garcimangosone D; guaijaverin; 2,6-dihydroxy-4-O-β-D-glucopyranosyl benzophenone; α-terpineol; psiguajdianone; psidial F; psidguajones A and B; asiatic acid; quercetin; quercetin-3-O-arabinoside; quercetin-3-O-sulphate; epicatechin; avicularin; quercetin 3-O-β-D-glucoside (isoquercetin); quercetin 3-O-β-D-galactoside (hyperin)	Ethanol extract of *P. guajava* leaves (at a dose of 200 and 400 mg/kg body weight) showed a significant reduction in the total number of stools, stool weight and average stool defecation rate in rats infected with enteropathogenic *E. coli* Admission of extract of *P. guajava* leaves at a dose of 50, 100 and 200 mg/kg body weight demonstrated a reduction in the incidence of diarrhoea in weaned piglets infected with enterotoxigenic *E. coli*	[48, 66]
	*Myristica fragrans* (20)	Trimyristin; myristicin; dihydrogualaretic acid	*M. fragrans* extract can inhibit prostaglandin synthesis in the colon mucosa, so it can be used as an antidiarrheal agent for patients with thyroid medullary carcinoma	[67]

Abbreviation: UR = use reports.

**Table 4:** Results of the informant's consensus factor (ICF) for several diseases related to mild COVID-19 symptoms.

Category of diseases	*N*_*ur*_	*N*_*t*_	ICF
Cough	176	39	0.78
Colds	106	29	0.73
Fever	144	43	0.71
Dizziness	104	30	0.72
Diarrhoea	137	22	0.85

**Table 5:** Side effects reported by informants after using medicinal plants for several diseases related to mild COVID-19 symptoms.

Family	Scientific name	Side effects (*n*)
Acanthaceae	*Andrographis paniculata* (Burm.f.) Nees	None (6); nauseous vomit (2)
	*Hemigraphis colorata* W.Bull	None (1)
	*Strobilanthes crispa* Blume	Nauseous vomit (1)

Amaranthaceae	*Amaranthus tricolour* L.	Nauseous vomit (1)

Amaryllidaceae	*Allium cepa* L.	None (9); nauseous vomit (1)
	*Allium sativum* L.	None (18); nauseous vomit (2); anaemia (1)
	*Allium tuberosum* Rottler ex Spreng.	None (8); nauseous vomit (2)

Anacardiaceae	*Mangifera indica* L.	None (1)

Annonaceae	*Annona muricata* L.	None (2); drowsiness (1); nauseous vomit (1)

Apiaceae	*Centella asiatica* (L.) Urb.	None (5)

Apocynaceae	*Alstonia scholaris* (L.) R. Br.	None (1)

Asteraceae	*Blumea balsamifera* (L.) DC.	None (4)
	*Vernonia amygdalina* Delile	None (1)

Basellaceae	*Anredera cordifolia* (Ten.) Steenis	None (1); nauseous vomit (1)

Caricaceae	*Carica papaya* L.	None (5); nauseous vomit (3)

Clusiaceae	*Garcinia* × *mangostana* L.	None (4)

Commelinaceae	*Commelina diffusa* Burm.f.	None (13); nauseous vomit (1)

Crassulaceae	*Bryophyllum pinnatum* (Lam.) Oken	None (13); nauseous vomit (2)

Cucurbitaceae	*Lagenaria siceraria* (Molina) Standl.	None (1)
	*Momordica charantia* L.	Nauseous vomit (2)
	*Momordica cochinchinensis* (Lour.) Spreng.	None (1)

Euphorbiaceae	*Acalypha indica* L.	Nauseous vomit (1)
	*Codiaeum variegatum* (L.) Rumph. ex A.Juss.	None (1)
	*Euphorbia hirta* L.	None (2)
	*Jatropha curcas* L.	None (30); nauseous vomit (4)
	*Jatropha gossypiifolia* L.	None (1)
	*Manihot esculenta* Crantz	None (2)
	*Melanolepis multiglandulosa* (Reinw. Ex Blume) Rchb. and Zoll.	None (2)

Fabaceae	*Erythrina subumbrans* (Hassk.) Merr.	None (1)
	*Senna tora* (L.) Roxb.	None (1); nauseous vomit (1)
	*Sesbania grandiflora* (L.) Pers.	None (10); others (1)
	*Tamarindus indica* L.	None (3)

Lamiaceae	*Clerodendrum japonicum* (Thunb.) Sweet	None (1)
	*Plectranthus amboinicus* (Lour.) Spreng.	None (4)
	*Plectranthus scutellarioides* (L.) R.Br.	None (40); nauseous vomit (6); laxative (1); others (1)
	*Ocimum americanum* L.	None (2)
	*Ocimum basilicum* L.	None (16); drowsiness (1); nauseous vomit (1)
	*Ocimum tenuiflorum* L.	None (3)
	*Orthosiphon aristatus* (blume) Miq.	None (7); nauseous vomit (2)

Lauraceae	*Cinnamomum verum* (J.Presl)	None (7)
	*Persea americana* Mill.	None (1)

Malvaceae	*Abelmoschus manihot* (L.) Medik.	None (1); nauseous vomit (1)
	*Hibiscus mutabilis* L.	None (1)
	*Hibiscus rosa-sinensis* L.	Nauseous vomit (1)

Meliaceae	*Aglaia odorata* Lour.	Nauseous vomit (1)

Menispermaceae	*Tinospora crispa* (L.) Hook. f. and Thomson	None (2); nauseous vomit (1)

Moraceae	*Artocarpus altilis* (Parkinson ex F.A.Zorn) Fosberg	None (3); drowsiness (2); Nauseous vomit (1)
	*Artocarpus heterophyllus* Lam.	None (2); nauseous vomit (1)

Moringaceae	*Moringa oleifera* Lam.	None (3); nauseous vomit (1)

Muntingiaceae	*Muntingia calabura* L.	None (7); drowsiness (1)

Musaceae	*Musa acuminata*	None (1)
	*Musa acuminata “lady finger”*	None (2)

Myristicaceae	*Myristica fragrans* Houtt.	None (22); nauseous vomit (1)

Myrtaceae	*Melaleuca leucadendra* (L.) L.	None (3)
	*Psidium guajava* L.	None (63); nauseous vomit (5); constipation (1)
	*Syzygium aromaticum* (L.) Merr. and L.M.Perry	None (10)
	*Syzygium cumini* (L.) Skeels	None (2)
	*Syzygium polyanthum* (Wight) Walp.	Nauseous vomit (1)

Oxalidaceae	*Averrhoa bilimbi* L.	None (10); nauseous vomit (5); others (1)
	*Averrhoa carambola* L.	None (1)

Phyllanthaceae	*Phyllanthus niruri* L.	None (2)

Piperaceae	*Piper betle* L.	None (11); nauseous vomit (2); drowsiness (1)
	*Piper nigrum* L.	None (3)

Poaceae	*Cymbopogon citratus* (DC.) Stapf	None (11); nauseous vomit (2)

Primulaceae	*Labisia pumila* (Blume) Fern.-Vill.	None (1)

Rhamnaceae	*Ziziphus spina-christi* (L.) Desf.	None (1)

Rubiaceae	*Morinda citrifolia* L.	None (4)
	*Uncaria gambir* (Hunter) Roxb.	None (1)

Rutaceae	*Citrus aurantiifolia* (Christm.) Swingle	None (19); nauseous vomit (2)
	*Citrus limon* (L.) Osbeck	None (16); gastric irritation (1); heartburn (1)
	*Citrus sinensis* (L.) Osbeck	None (1)

Theaceae	*Camellia sinensis* (L.) Kuntze	None (1)

Thymelaeaceae	*Phaleria macrocarpa* (Scheff.) Boerl.	None (1)

Verbenaceae	*Lantana camara* L.	None (1)

Zingiberaceae	*Alpinia galanga* (L.) Willd.	None (1)
	*Curcuma longa* L.	None (56); nauseous vomit (1)
	*Curcuma zanthorrhiza* Roxb.	None (8)
	*Curcuma zedoaria* (Christm.) Roscoe	None (1)
	*Kaempferia galanga* L.	None (17); nauseous vomit (2)
	*Zingiber montanum* (J.Koenig) Link ex A.Dietr.	None (1)
	*Zingiber officinale* Roscoe	None (59); nauseous vomit (6); heartburn (2)
	*Zingiber officinale* var. *rubrum*	None (3)

*Note: n* = number of informants who reported side effects of medicinal plants.

## Data Availability

The data used to support the findings of this study are available from the corresponding author upon request.
